# A bloody surprise: rare presentation of pituitary apoplexy with epistaxis—a case report

**DOI:** 10.1093/bjrcr/uaag007

**Published:** 2026-04-02

**Authors:** Kevin Jun Hui Kow, Jaya Sri Konakanchi, Vaanathi Paulvannan, Jooly Joseph

**Affiliations:** Department of Radiology, University Hospitals of North Midlands NHS Trust, Stoke-on-Trent ST46QZ, United Kingdom; Department of Neurosurgery, University Hospitals of North Midlands NHS Trust, Stoke-on-Trent ST46QZ, United Kingdom; Department of Radiology, University Hospitals of North Midlands NHS Trust, Stoke-on-Trent ST46QZ, United Kingdom; Department of Radiology, University Hospitals of North Midlands NHS Trust, Stoke-on-Trent ST46QZ, United Kingdom

**Keywords:** pituitary apoplexy, epistaxis, tumour

## Abstract

Pituitary apoplexy (PA) is an endocrine emergency caused by haemorrhage or infarction of the pituitary gland, often occurring in patients with known tumours. Classical presentation includes headache, visual disturbances, and cranial nerve palsies. We describe a rare presentation of PA with epistaxis in a 71-year-old man with a known non-functioning pituitary macroadenoma. On admission, he had acute confusion, worsening headache, and left-sided epistaxis with signs of Cranial Nerve (CN) III palsy and reduced consciousness. Initial Computerized Tomography (CT) showed a stable macroadenoma without acute changes. Laboratory evaluation showed panhypopituitarism and features of vasopressor deficiency. Rapid clinical deterioration prompted repeat CT and Magnetic Resonance Imaging (MRI), which demonstrated interval enlargement of the adenoma with haemorrhage and mass effect. MRI also revealed a catastrophic left middle cerebral artery infarct secondary to M1 segment thrombosis. Despite intensive care support, the patient failed to recover neurologically and passed away. Our case report describes the rare occurrence of epistaxis in PA, due to tumour extension into the sphenoid sinus and erosion of the sellar floor. We emphasize the importance of an early MRI and endocrinology review for timely diagnosis and management. We underscore the need to consider PA in patients with known adenomas and acute neurological decline, even with atypical symptoms. Our case report adds to the limited literature on epistaxis as a presenting sign of PA and associated high morbidity.

## Clinical presentation

**Figure 1.**
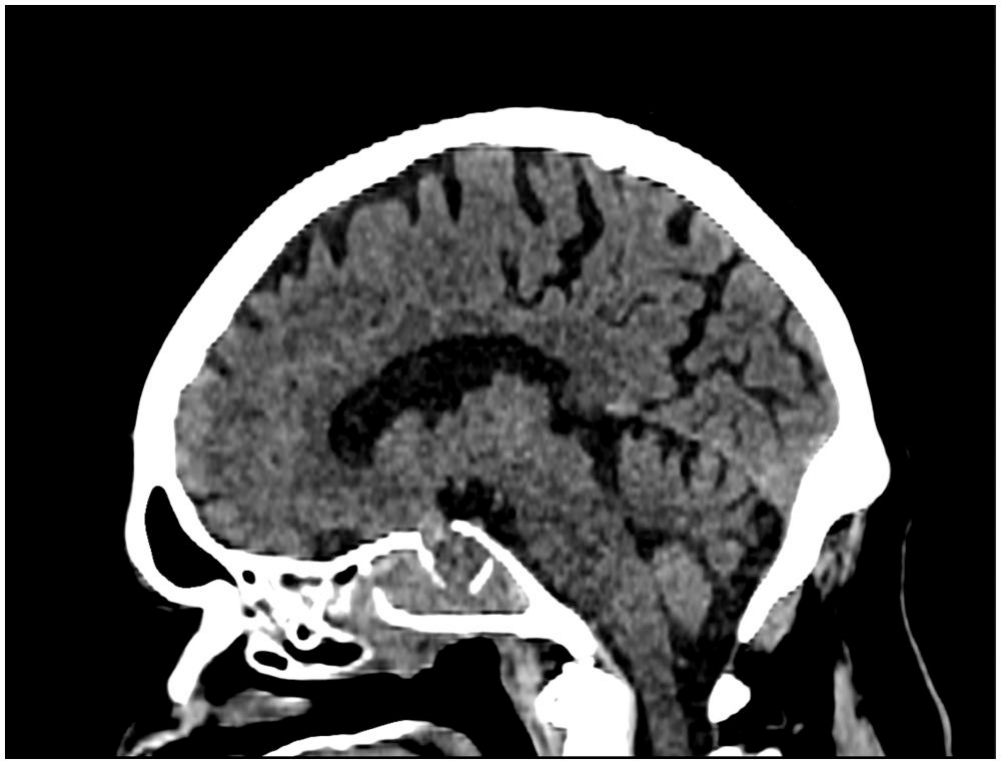
Pituitary adenoma with no acute features and a bony defect in the sella.

A 71-year-old gentleman presented with acute confusion, worsening headaches, and epistaxis. A week before the presentation, he was treated for a lower respiratory tract infection with antibiotics, yet he continued to decline cognitively. He had a background of bipolar disorder (on sodium valproate, previously lithium) and an incidental non-functioning pituitary macroadenoma that was diagnosed on CT brain 2 years ago. Given the lack of symptoms, the pituitary tumour was managed conservatively. However, the initial imaging demonstrated that it had extended into the clivus, sphenoid sinus, and bilateral cavernous sinuses. There was also a bony defect within the sellar floor into the sphenoid sinus (Figure 1).

Examination revealed confusion with a Glasgow Coma Scale (GCS) of 14, ptosis of the left eye, and a fixed and dilated left pupil of 5 mm (signs of CN III palsy). The range of eye movements was difficult to assess, suggesting other CN involvement. The left nostril demonstrated evidence of epistaxis with dry clotted blood, while the right nostril was clear.

## Differential diagnosis

Given the acuity of symptoms and prior treatment for respiratory tract infection, the differential diagnosis focused primarily on infective pathology, including meningitis, encephalitis, delirium, and sepsis. The nature of the headache also raised suspicion of cerebral venous sinus thrombosis (CVST). Given the known history of non-functioning pituitary macroadenoma with CN III palsy, differentials also included cavernous sinus thrombosis and pituitary apoplexy (PA).

## Investigations/imaging findings

Initial lab findings revealed raised inflammatory markers, which led to a suspicion of sepsis, and treatment was commenced using empirical antibiotics. As the patient had headaches and confusion, a CT head was performed, which showed no acute abnormality and a stable appearance of the pituitary macroadenoma (Figure 1). The working diagnosis was for a primary central nervous system (CNS) infection with a differential of CVST. Unfortunately, a lumbar puncture was not done. The patient then had a CT intracranial venogram, which was negative.

**Figure 2.**
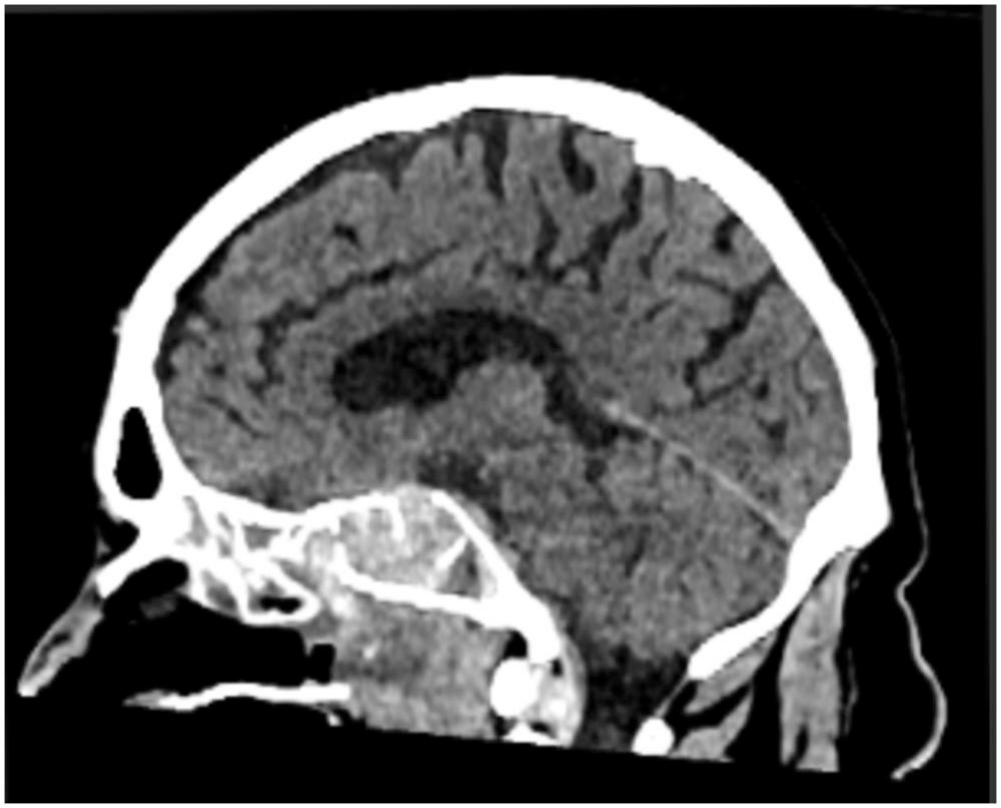
High-density material in the sella extending into the sphenoid sinus.

Unfortunately, the patient deteriorated further with recurrent epistaxis and a drop in GCS from 13 to 5, requiring intubation. A subsequent CT head was obtained, which showed a significant increase in the size of the pituitary adenoma with a new mass effect into the suprasellar cistern. The high-density material within the tumour was thought to represent a small-volume haemorrhage (Figure 2). Investigations done in view of suspected pituitary dysfunction revealed a panhypopituitary profile as seen in Table 1.

**Table 1:** 

Hormone	Results	Normal range
Luteinizing hormone (LH)	0.3 IU/L	1.5–9.3 IU/L
Follicle stimulating hormone (FSH)	1.4 IU/L	1.4–18.1 IU/L
Thyroid Stimulating hormone (TSH)	0.11 mlU/L	0.38–5.33 mlU/L
Free T4	14.4 pmol/L	11.5–22.7 pmol/L
Testosterone	0.3 nmol/L	6.5–23.7 nmol/L
Prolactin	21 µg/L	20 µg/L
Insulin like growth factor 1(IGF-1)	5 ng/mL	15-307 ng/mL
Cortisol	>2000 nmol/L	138 to 635 nmol/L
Growth hormone	12 µg/L	Minimum 10 ug/L

The patient also demonstrated persistent hypernatraemia and diabetes insipidus (DI), initially attributed to nephrogenic DI secondary to his previous use of lithium for bipolar disorder. However, given the concurrent hypopituitarism, central DI was subsequently considered more likely.

**Figure 3.**
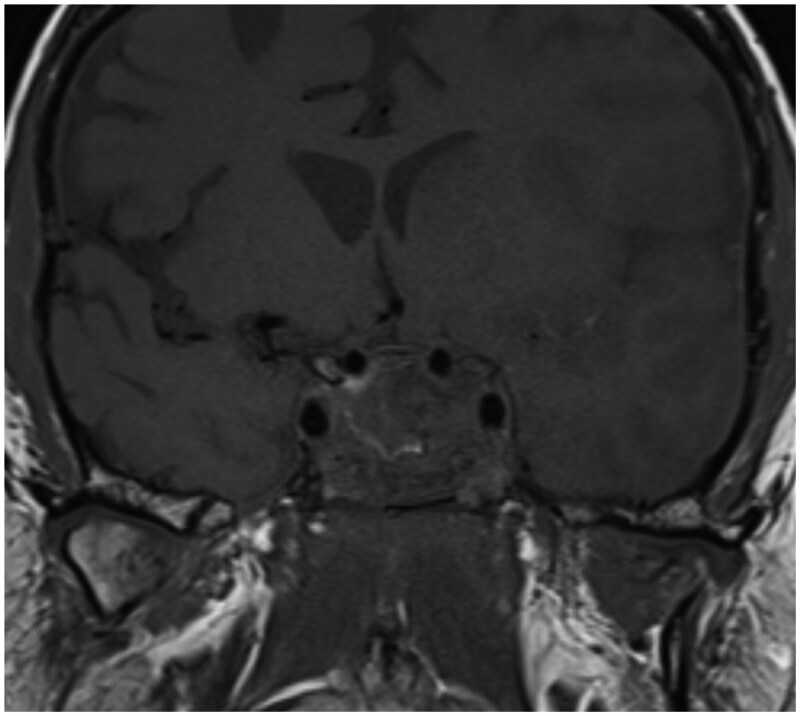
T1 high intensity within the sella is suggestive of haemorrhage.

**Figure 4.**
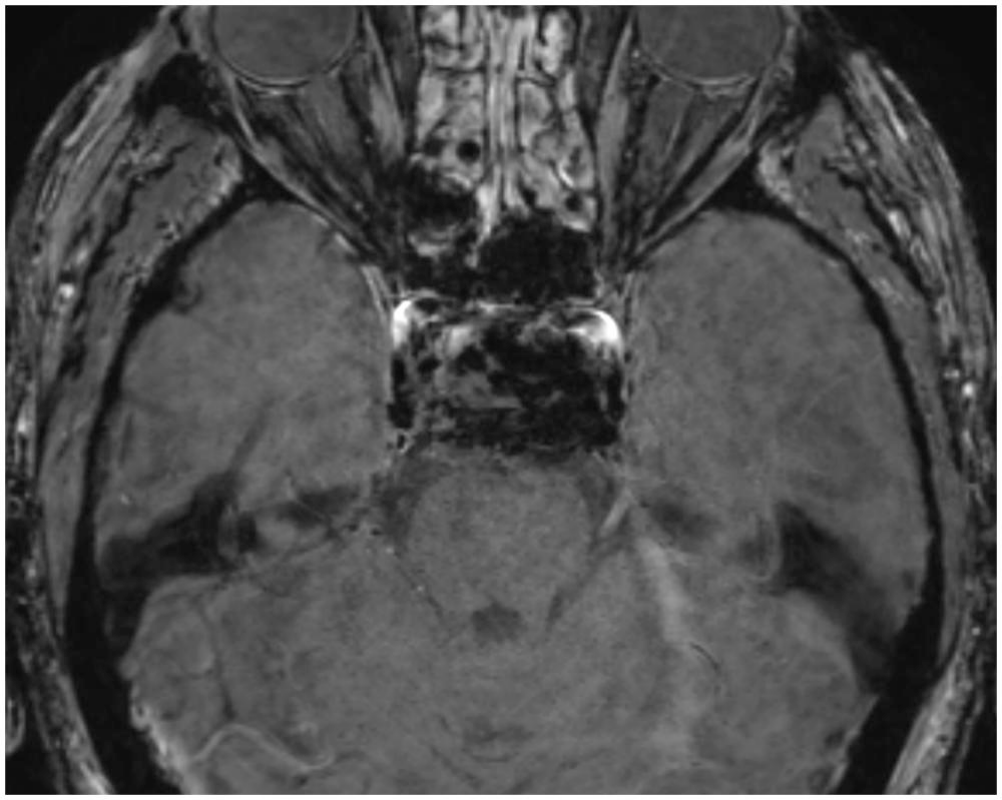
SWI blooming artefact within sella in keeping with haemorrhage.

**Figure 5.**
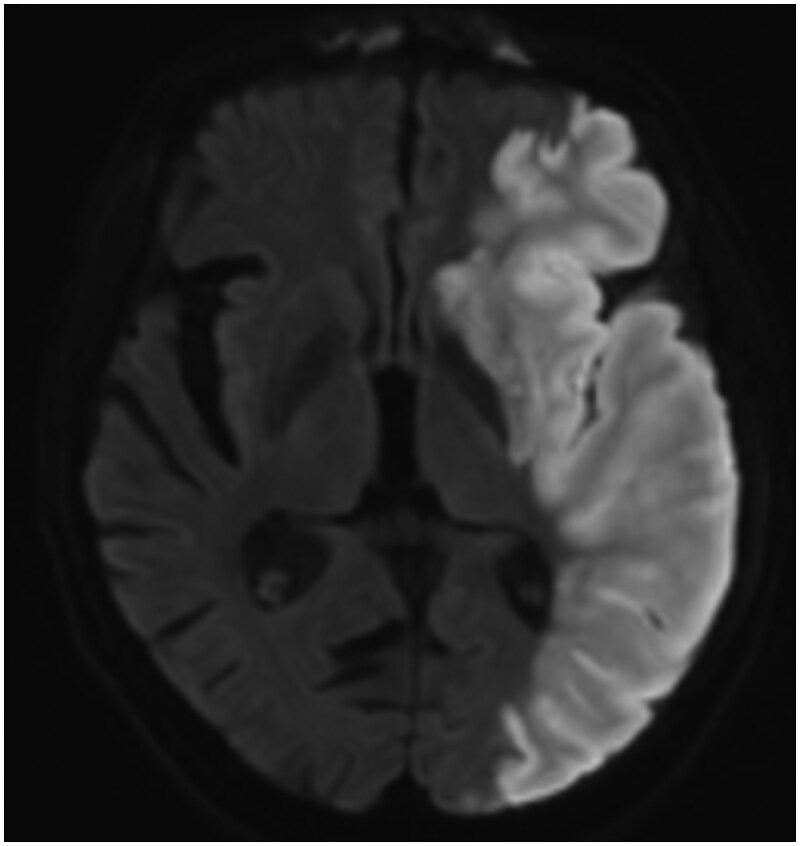
Left MCA territory infarct demonstrated by the DWI signal abnormality.

An MRI was requested to confirm the diagnosis of PA and to assess any further cranial nerve involvement. Following abnormalities noted on the initial sequences, additional stroke sequences, including MR angiography (MRA), were obtained. These confirmed the diagnosis of PA and also demonstrated an infarct in the left middle cerebral artery (MCA) territory (Figures 3-5). MRA demonstrated a thrombus within the left M1 segment.

## Learning points

PA can also present with atypical, vague symptoms as seen in our patient. Especially in patients with known adenomas, the possibility of apoplexy must be considered if they present with neurological symptoms. Prompt imaging and hormone assay are essential for appropriate management.

## Treatments

During the initial days of admission, our patient was treated with antibiotics (ceftriaxone, metronidazole, and aciclovir) under suspicion of infection. After intubation and admission to intensive care, despite ongoing hormone supplementation, he continued to deteriorate further. Neurosurgery referral was sought, but given the ongoing complex clinical picture with poor GCS, he was deemed not fit for any surgical intervention. Even when off sedation, he demonstrated no motor response (GCS 3 for 3-4 days). After further discussions between the neurosurgical, endocrinology, and intensive care teams, including the patient’s family, the decision was made to prioritise patient comfort and not pursue any further active intervention.

## Outcome, follow-up, and discussion

Unfortunately, the patient passed away secondary to the large territory infarct, causing severe neurological compromise.

Numerous factors can precipitate an apoplexy, such as angiographic procedures, surgeries, trauma, anticoagulation therapies, and even dynamic tests.^1^ In general, nonfunctioning adenomas tend to be associated with apoplexy, though this could be due to late presentation and larger size.^2^ As the tumour grows larger, it has increased metabolic demands while also outgrowing its blood supply, resulting in necrosis, which may be associated with haemorrhage.^2^

The classic clinical scenario for presentation includes headache, vomiting, visual defects, and extraocular palsies.^3^ There have been three published cases of apoplexy associated with epistaxis, as per our literature review. Peng et al described a patient having apoplexy due to an associated vascular aneurysm.^4^ Teasdale et al described a case of epistaxis due to recurrent PA.^5^ Keane et al reported a case of epistaxis due to apoplexy that presented as meningitis.^6^ Our patient had a similar history that was suggestive of an infective aetiology with epistaxis.

MRI is the diagnostic imaging of choice.^3^ While a CT is usually the first-line imaging, it is not as sensitive and may show sellar haemorrhage in only 20%-30% of cases.^3^ This is probably the reason for the initial normal CTH in our patient.

Early infarcts may not be demonstrable on T1- and T2-weighted sequences but are usually detected on diffusion-weighted imaging (DWI).^3^ In cases of haemorrhage, the signal characteristics vary with time. In the acute phase, haemorrhage typically appears isointense to hypointense on T1 and markedly hypointense on T2 due to the presence of intracellular deoxyhaemoglobin. During the early subacute phase, it becomes hyperintense on T1 and hypointense on T2 (intracellular methemoglobin), while in the late subacute phase it appears hyperintense on both T1 and T2 (extracellular methemoglobin).^2^ Following gadolinium administration, peripheral enhancement may be observed–the so-called ‘pituitary rim sign’. Gadolinium contrast may show peripheral enhancement in keeping with the ‘pituitary rim sign’.^2^ Associated findings may include thickening of the sphenoid sinus mucosa and air-fluid levels.

This can also be associated with the thickening of the sphenoid sinus mucosa with air-fluid levels.^2^ In our patient, the MRI demonstrates some marginal high T1 and a heterogeneous T2 signal (Figure 3). There was a sellar blooming artefact on the susceptibility-weighted imaging (Figure 4). MRA done at the same time demonstrates a left M1 thrombus with extensive left hemisphere diffusion restriction in keeping with an acute infarct (Figure 5).

Ahn et al published a case report about a patient who developed cerebral ischemia following a PA.^7^ The patient had undergone surgical resection and angioplasty, which showed some improvement.^7^ Overall, the findings in their case were in keeping with vascular compression secondary to the pituitary haematoma.^7^ In our patient, the MRA demonstrated a patent ICA intracranially with an abrupt cut-off at the left MCA M1 segment from a thrombus. It was postulated that this could be a prothrombotic state secondary to the general inflammation of the PA.

Treatment is highly variable and includes either conservative management with hormone replacement or surgical management with tumour resection. The 2010 UK guidelines have established criteria for surgical and conservative management.^8^

The initial management is medical with steroid replacement to avoid secondary adrenal insufficiency.^8^ Patients with deteriorating visual symptoms tend to benefit more from surgery.^8^ Patients who undergo conservative treatment may show a spontaneous shrinkage in the size of the tumour in the following weeks.^8^ The 2010 UK guidelines have established criteria for surgical and conservative management.^8^

We stress the importance of early MRI imaging and prompt endocrinology evaluation for accurate diagnosis and effective management. This case adds to the limited literature on epistaxis as an unusual but significant presenting symptom of PA, emphasising the need to consider it in patients with known pituitary adenomas and sudden neurological decline.

## References

[1] Johnston PC , Hamrahian AH, Weil RJ, Kennedy L. Pituitary tumor apoplexy. J Clin Neurosci. 2015;22:939-944. 10.1016/j.jocn.2014.11.023 25800143

[2] Muthukumar N. Pituitary apoplexy: a comprehensive review. Neurol India. 2020;68:S72-S78. 10.4103/0028-3886.287669 32611895

[3] Briet C , Salenave S, Bonneville JF, Laws ER, Chanson P. Pituitary apoplexy. Endocr Rev. 2015;36:622-645. 10.1210/er.2015-1042 26414232

[4] Peng Z , Tian D, Wang H, et al Epistaxis and pituitary apoplexy due to ruptured internal carotid artery aneurysm embedded within pituitary adenoma. Int J Clin Exp Pathol. 2015;8:14189-14197. 26823732 PMC4713518

[5] Teasdale S , Hashem F, Olson S, Ong B, Inder WJ. Recurrent pituitary apoplexy due to two successive neoplasms presenting with ocular paresis and epistaxis. Endocrinol Diabetes Metab Case Rep. 2015;2015:140088. 10.1530/EDM-14-0088 25755879 PMC4322370

[6] Keane JR. Pituitary apoplexy presenting with epistaxis. J Neuro-Ophthalmol. 1984;4:7-8. 6233314

[7] Ahn JM , Oh HJ, Oh JS, Yoon SM. Pituitary apoplexy causing acute ischemic stroke: which treatment should be given priority. Surg Neurol Int. 2020;11:113. 10.25259/SNI_82_2020 32494388 PMC7265385

[8] Rajasekaran S , Vanderpump M, Baldeweg S, et al UK guidelines for the management of pituitary apoplexy. Clin Endocrinol (Oxf). 2011;74:9-20. 10.1111/j.1365-2265.2010.03913.x 21044119

